# Influence of the Chemical Form of Antimony on Soil Microbial Community Structure and Arsenite Oxidation Activity

**DOI:** 10.1264/jsme2.ME17182

**Published:** 2018-06-09

**Authors:** Takafumi Kataoka, Satoshi Mitsunobu, Natsuko Hamamura

**Affiliations:** 1 Faculty of Marine Science and Technology, Fukui Prefectural University Gekuen-Cho 1–1 Obama, Fukui, 917–0003 Japan; 2 Center for Marine Environmental Studies (CMES), Ehime University 2–5 Bunkyo-cho, Matsuyama, Ehime 790–8577 Japan; 3 Department of Bioresources, Faculty of Agriculture, Ehime University 3–5–7 Tarumi, Matsuyama, Ehime 790–8566 Japan; 4 Department of Biology, Faculty of Science, Kyushu University 744 Motooka, Nishiku, Fukuoka 819–0395 Japan

**Keywords:** soil microbial community, arsenite oxidase gene (*aio*), multiple metalloid contamination, antimony, advective flow cultivation

## Abstract

In the present study, the influence of the co-contamination with various chemical forms of antimony (Sb) with arsenite (As[III]) on soil microbial communities was investigated. The oxidation of As(III) to As(V) was monitored in soil columns amended with As(III) and three different chemical forms of Sb: antimony potassium tartrate (Sb[III]-tar), antimony(III) oxide (Sb_2_O_3_), and potassium antimonate (Sb[V]). Soil microbial communities were examined qualitatively and quantitatively using 16S rDNA- and arsenite oxidase gene (*aioA*)-targeted analyses. Microbial As(III) oxidation was detected in all soil columns and 90–100% of added As(III) (200 μmol L^−1^) was oxidized to As(V) in 9 d, except in the Sb(III)-tar co-amendments that only oxidized 30%. 16S rDNA- and *aioA*-targeted analyses showed that the presence of different Sb chemical forms significantly affected the selection of distinct As(III)-oxidizing bacterial populations. Most of the 16S rRNA genes detected in soil columns belonged to *Betaproteobacteria* and *Gammaproteobacteria*, and some sequences were closely related to those of known As(III) oxidizers. Co-amendments with Sb(III)-tar and high concentrations of Sb_2_O_3_ significantly increased the ratios of *aioA*-possessing bacterial populations, indicating the enrichment of As(III) oxidizers resistant to As and Sb toxicity. Under Sb co-amendment conditions, there was no correlation between *aioA* gene abundance and the rates of As(III) oxidation. Collectively, these results demonstrated that the presence of different Sb chemical forms imposed a strong selective pressure on the soil bacterial community and, thus, the co-existing metalloid is an important factor affecting the redox transformation of arsenic in natural environments.

Arsenic (As) and antimony (Sb) are naturally occurring toxic elements that are regarded as priority pollutants of interest by the United States Environmental Protection Agency. Although the concentrations of these toxic elements in natural systems are generally low (~15 mg kg^−1^ As and <1 mg kg^−1^ Sb in soils [12]), elevated levels of As and Sb are released via natural processes and human activities. Both As and Sb often co-occur in the environment and exhibit similar geochemical properties and toxicological effects depending on their chemical forms and oxidation states (12). Arsenic and antimony may exist in four oxidation states (–III, 0, III, and V), while they are mainly found in two oxidation states, trivalent (III) and pentavalent (V), in natural systems. The trivalent forms, As(III) and Sb(III), are highly reactive with thiol-containing proteins and are considered to be more toxic than As(V) and Sb(V) (12, 53). Since As(V) has stronger affinity to minerals than As(III) (44), the oxidation of trivalent species is an important process for the immobilization and detoxification of arsenic.

Microorganisms have developed mechanisms to tolerate these metalloids and mediate the redox transformation of As and Sb. Microbial As redox transformations have been studied in detail in phylogenetically diverse groups of bacteria. The oxidation of As(III) coupled to O_2_ reduction is catalyzed by arsenite oxidase (Aio) and has been described in numerous heterotrophic bacteria as well as in some chemoautotrophs (3, 9, 41, 45). Phylogenetically diverse As(III)-oxidizing bacteria have been isolated from As-contaminated environments (45), and shown to play an important role in rapid As(III) oxidation *in situ*. Regarding Sb, recent advances revealed the involvement of microorganisms in Sb redox transformation (27); however, few studies have focused on its effects on the microbial community and catabolic potential. The main chemical form of Sb(III) in the natural environment is antimony trioxide (Sb_2_O_3_), a poorly soluble mineral form that is considered to be non- or weakly toxic to biota (15, 36). In contrast, the highly soluble organic form of Sb(III), antimony tartrate, was reported to exhibit stronger toxicity than the mineral form to soil microbiota (2, 16).

Microbial communities are affected as a result of adaptation to a short- or long-term exposure to heavy metals and metalloids (8, 28, 49). The resistance level for variable concentrations of As(III) or Sb(III) differs among As(III)-oxidizing (6) or Sb(III)-oxidizing bacterial strains (26, 43). A previous study showed that the difference in co-contaminating metal(loid)s affected not only bacterial As resistance, but also microbial community structures and activities (49). Although As and Sb often co-occur in a contaminated environment, the co-contamination effects of two metalloids on the microbial community have yet to be clarified (50).

The main objective of the present study is to evaluate the effects of Sb co-contamination in addition to As on the soil microbial community and its As(III) oxidation activity. We hypothesized that co-contamination with various chemical forms of Sb may exert different effects on the soil bacterial community composition, thereby resulting in changes in microbiological As(III) oxidation. The oxidation rates of As(III) to As(V) and changes in the microbial community were monitored in soil columns amended with As(III) and three different chemical forms of Sb: antimony(III) potassium tartrate (Sb[III]-tar), antimony(III) oxide (Sb_2_O_3_), and antimonate (Sb[V]). The results of the present study revealed the complex inhibitory effects of co-existing metalloids on the microbial community, which may affect the redox transformation of metalloid contaminants in natural environments.

## Materials and Methods

### Soil sample collection

Soil samples were collected from a depth of 0 to 12 cm, excluding the organic layer, at the Ichinokawa antimony mine, Ehime, Japan (33° 53′ 355″ N; 133° 12′ 848″ E). Soil was collected using sterile techniques, transported on ice to the laboratory, passed through a 2-mm sieve, and stored field moist at 4°C. The pH of the sampled soil was measured as 5.6 by dissolving in a 1:1 volume of distilled water on site. The concentrations of water-soluble As and Sb were determined to be 0.09±0.02 and 0.16±0.02 mg kg^−1^, respectively, using an inductively coupled plasma optical emission spectrometer (ICP-OES, Optima 7300 DV; PerkinElmer) as previously described (18).

### Soil column experiments

Column experiments were conducted with an advective flow system using a sterile plastic syringe (length=90 mm, diameter=22 mm) packed with a mixture of 5% (w/w) soil and 95% (w/w) acid-washed, autoclaved quartz sand (50–70 mesh, Sigma-Aldrich, St. Louis, MO, USA) for a total mass of 56.5–60.9 g (the bulk density was *ca.* 1.65–1.71 g mL^−1^). In order to maintain aerobic conditions and unsaturated flow inside the column, filter-sterilized (0.2 μm) air was supplied through the bottom of the column using an air pump at a rate of 25.6 mL min^−1^. The columns were supplied with an autoclaved influent through a filter with a pore size of 0.22 μm from the bottom of the columns with continuous flow at a rate of 1.5 mL h^−1^ (1.05 column volume d^−1^; pore water velocity 0.39 cm h^−1^).

The influent was formulated to simulate the soil solution (30), which contained NH_4_NO_3_ (1.25 mM), CaSO_4_ (0.03 mM), MgCl_2_ (0.02 mM), KH_2_PO_4_ (0.01 mM), KOH (1.25 mM), FeCl_3_ (0.02 mM), and FeCl_2_ (0.02 mM) supplemented with trace elements: MnCl_2_ (0.03 mM), ZnSO_4_ (0.013 mM), H_3_BO_3_ (0.02 mM), Na_2_MoO_4_ (0.009 mM), NiCl_2_ (0.002 mM), CuCl_2_ (0.002 mM), and CoCl_2_ (0.002 mM) (52) with 0.02% (w/v) yeast extract as a carbon source. The pH of the influent solution was adjusted to 6.8. In addition to 0.2 mM of As(III) (as NaAsO_2_), three chemical forms of antimony were added: antimonate as K(Sb[OH]_6_) (0.4 mM); antimonite as a potassium tartrate salt (C_8_H_3_K_2_O_12_Sb_2_ [0.15 mM]) or as Sb_2_O_3_ (0.012–0.143% w/total column soil weight corresponding to concentrations of 4.1–46 Sb mmol L^−1^ of the water content in the soil column) (Table 1). Biotic controls were conducted i) in the presence of As(III) without the addition of antimony (As only control), or ii) in the presence of As(III) with potassium-L-tartrate (KC_4_H_5_O_6_ [0.15 mM]) to examine the inhibitory effects of tartrate on As(III) oxidation activity (tartrate control). In addition, an abiotic control was conducted with autoclaved soil that was prepared as follows: a preserved soil sample was sieved using a 2-mm square mesh to remove large particles, sterilized three times by autoclaving (121°C, 1 h) at an interval of 24 h.

During the duration of the experiment, the effluent was collected daily in a sterile polyethylene tube and weighed to calculate the flow rate. For the measurement of dissolved As and Sb species, an aliquot (1 mL) was immediately frozen at −80°C and stored until subjected to measurements with ICP-OES. As(III) and Sb transformation activities were monitored by ICP-OES for total and pentavalent species after the borohydride reduction-based liberation of Sb(III) and As(III) as stibine and arsine gases, respectively, as described previously (24, 31). The detection limit for arsenic and antimony was >0.05 ppm.

### Nucleic acid extraction and PCR amplification of 16S rRNA and *aioA* genes

For the molecular analysis, the column content was collected at each selected time point by sacrificing the whole column, *ca.* 0.5 g of the quartz sand-soil mixture was placed into 2-mL tubes and immediately frozen at −80°C until genomic DNA extraction. Total DNA for the molecular analysis was extracted from *ca.* 0.5 g of the subsampled column content using the Power Soil DNA Isolation kit^®^ (Mo Bio Laboratories, Carlsbad, CA, USA). The concentration of the DNA solution was quantified using PicoGreen^®^ (Invitrogen, Carlsbad, CA, USA) with the SYBR^®^ Green detection system on a CFX96™ optical reaction module (Bio-Rad Laboratories, Hercules, CA, USA).

For denaturing gradient gel electrophoresis (DGGE), bacterial 16S rRNA gene fragments were PCR-amplified using a Bacteria-domain specific primer (1070F) and universal primer (1392R) incorporating a GC clamp (11), followed by the separation of PCR products using DGGE as described previously (17). The *aioA* gene was PCR-amplified using the non-degenerated primer set *aoxB*M1-2F-ND and *aoxB*M2-1R-ND attached to the GC clamp as described previously (38). DGGE gels were stained with SYBR^®^ Gold (Invitrogen), visualized, and photographed with an imaging system (ChemiDoc™, Bio-Rad). The DNA sequences of the prominent DGGE bands were determined as described previously (11). Sequences were compared to the GenBank database using BLAST, and for 16S rRNA genes, sequences were aligned against SILVA alignment (SSU Ref NR 110; [37]) with the Fast Aligner tool on ARB software (29). For the phylogenetic analysis of the *aioA* gene, a reference phylogenetic tree was constructed with the amino acid sequences (351 residues) of known AioA sequences using ARB. The nucleotide sequences obtained in this study have been deposited in the DDBJ nucleotide sequence database under accession numbers AB974172–AB974188 and AB974215–AB974226.

### Quantification of 16S rRNA and *aioA* gene abundance

Real-time quantitative PCR (qPCR) was performed in order to quantify the copy numbers of 16S rRNA (46) and *aioA* genes (38, 39) as previously described. qPCR was performed in triplicate of 20 μL total volume containing 0.5 ng extracted DNA, primer-specific concentration of MgCl_2_ as described previously (38, 39, 46), and 1× SSofast™ Eva Green^®^ Supermix (Bio-Rad). Thermocycling conditions were as follows: after heating at 98°C for 2 min, primer-specific cycles of 98°C for 5 s, and annealing and elongation at 56°C for 15 s followed by melting curve detection. For the quantitative enumeration based on the gene copy number, standard curves were constructed from a ten-fold serial dilution between 1.0×10^2^ and 1.0×10^8^ copies per reaction of the PCR amplicon generated using the M13 primer set from a plasmid (pCR^®^ 4.0; Invitrogen) containing the target bacterial 16S rRNA gene or *aioA* gene sequences.

### Statistical analysis

A multiple scaling bootstrap clustering analysis of DGGE banding patterns was conducted by detecting the DGGE band showing more than 0.1% of total intensity using the densitometry application of ImageJ software, and banding patterns were converted into a binary matrix where 1 and 0 were given when a band was present and absent, respectively. For the clustering analysis, DGGE analyses including two to three time points per treatments were used (Fig. S3). The band-pattern dissimilarity between pairwise stations was calculated by the Jaccard index (S_Jaccard_):

$${\text{S}}_{\text{Jaccard}}={\text{N}}_{\text{AB}}/({\text{N}}_{\text{A}}+{\text{N}}_{\text{B}}-{\text{N}}_{\text{AB}})$$

where N_AB_ is the number of bands common to DGGE lanes A and B, and N_A_ and N_B_ are the total numbers of bands in lanes A and B, respectively. Unweighted pairwise grouping with mathematical averages (UPGMA) was employed for the clustering of samples with 2,000 multiscale bootstrap resamplings using the package pvclust ver. 1.2 (47) in software R.

An analysis of variations in the ratio of the copy numbers of the *aioA* gene to the 16S rRNA gene among treatments was tested using a one-way ANOVA followed by Tukey’s post-hoc test in software R (ver. 2.14).

## Results and Discussion

### Effects of Sb co-contamination on soil microbial As(III) oxidation

**Fig. 1:**
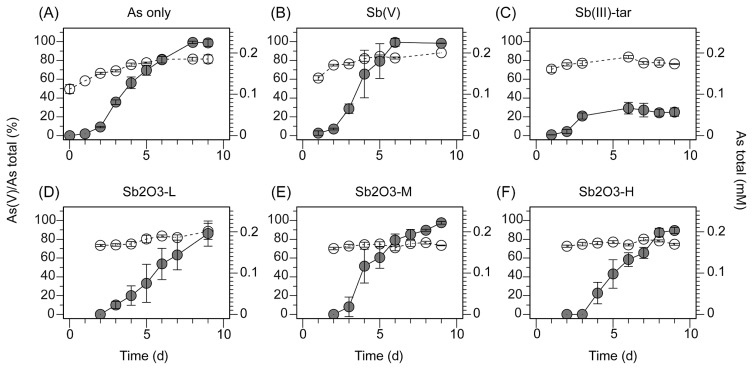
The production of arsenate, As(V), and concentration of total As in the effluent from column experiments. Closed circles represent the percentage of As(V) produced from the oxidation of As(III) to the total As concentration, and open circles represent total As concentrations (mM) in the effluent. Experimental conditions are described in Table 1. Each point represents the mean of at least triplicate soil columns; error bars represent the standard error, and where absent, error bars are smaller than the symbol size.

Arsenite oxidation was detected in As(III)-amended soil columns after day 3 and the concentration of As(V) in the effluent reached 185.0±4.5 μmol L^−1^ on day 8, as 92.5% of added As(III) was oxidized to As(V) (Fig. 1A). In contrast, less than 18.0±7.5 μmol of As(V) was detected in the effluent of sterilized controls over the same incubation period (data not shown). Consequently, the rapid oxidation of As(III) observed in the non-sterile columns was microbially mediated. The rate of microbial As(III) oxidation observed in the present study was comparable to the rates previously observed with similar soil column assays (*i.e.*, 128~158 μmol L^−1^ d^−1^ [23] and ~75 μmol L^−1^ d^−1^ [31]).

Among soil columns co-amended with various Sb chemical forms, the co-amendments with Sb(V) (Fig. 1B) and Sb_2_O_3_ (Fig. 1D–F) did not affect overall As(III) oxidation activity, and 90~100% of added As(III) was oxidized by day 9. However, a slightly slower increase in As(III) oxidation activity was observed in the Sb_2_O_3_-L and -H treatments (*i.e.*, 55~60% in these treatments and 75% in the As(III)-amended control were oxidized on day 6). In contrast, the co-amendment with Sb(III)-tar markedly inhibited As(III) oxidation after day 3 and As(III) oxidation remained at only ~30% of the added As concentration, corresponding to an As(V) concentration of ~43.1±6.5 μmol L^−1^ in the effluent, until the end of the incubation period (Fig. 1C). In all treatments, the rate of As(III) oxidation remained unchanged after day 10 for more than 20 d (data not shown). In order to eliminate the possibility of tartrate being inhibitory to As(III) oxidation, the soil column was also amended with As(III) and potassium tartrate. The tartrate co-amendment showed a similar pattern of As(III) oxidation activity as the As(III)-amended control (Fig. S1), confirming that Sb(III) provided as K_2_Sb_2_(C_4_H_2_O_6_)_2_ rather than potassium tartrate exerted inhibitory effects in the Sb(III)-tar treatment.

In contrast to the rapid microbial As(III) oxidation observed in the soil columns, Sb redox transformation was not apparent in any of the Sb co-amendments (Fig. S2). This result indicated that the As(III)-oxidizing population established in soil columns was unable to concomitantly oxidize Sb(III), which was consistent with our previous findings on the isolation of As(III)-oxidizing bacteria incapable of Sb(III) oxidation from antimony mine tailing soils (18). Some of the reported Sb(III)-oxidizing bacteria were capable of oxidizing As(III), and arsenite oxidase (Aio) in *Agrobacterium tumefaciense* strain 5A was shown to oxidize Sb(III) (51). Terry *et al.* also reported that *Variovorax paradoxus* strain IDSBO-4 was able to aerobically oxidize Sb(III) and As(III) concomitantly and the rate of As(III) oxidation in the co-amendment was nearly two-fold that of the culture amended with As(III) only (48). In contrast, these Sb(III)-oxidizing bacteria, previously isolated from the same mine soil used in this study (18), were unable to grow in the presence of As(III) and Sb(III) (unpublished data), suggesting that Sb(III)-oxidizing bacteria in soil columns are sensitive to As toxicity. Some of the Sb(III)-oxidizing bacteria were incapable of oxidizing As(III), suggesting the presence of distinct mechanisms for As(III) and Sb(III) oxidation (18, 43).

In the Sb(III)-tar treatment, the concentration of total soluble Sb in the effluent increased from 33.8±1.4 μmol L^−1^ on day 3 to 94.4±10.5 μmol L^−1^ on day 9 (Fig. S2C), while the concentration of Sb(V) in the effluent remained at less than 30 μmol L^−1^ during the incubation period. Sb(III) in the influent may have been initially retained in the soil column by sorption to the mineral or organic phase (22, 25), and released into the effluent after exceeding the sorption capacity. In the effluent of all Sb_2_O_3_ co-amendments, low levels of soluble Sb (*i.e.*, 31.5±8.8 μmol L^−1^) were also constantly detected, mostly as Sb(V), despite a 10-fold difference in the concentrations of added Sb_2_O_3_ between the L and H treatments. This may be explained by the abiotic oxidation of Sb_2_O_3_ and release of soluble Sb(V). Consequently, the higher toxicity of the tartrate form of Sb(III) than the mineral oxide (*i.e.*, Sb_2_O_3_) on soil microorganisms may have been caused by the bio-available soluble form of Sb(III). Although the toxic effects of Sb on soil microbial communities are relatively unknown, the present results are consistent with previous findings showing that Sb(III) was more toxic than Sb(V) to culturable soil microbial populations and enzyme activities (50), and that the soluble tartrate form of Sb exhibited higher toxicity than the mineral oxide (Sb_2_O_3_) on soil microbial respiration activities (16).

### Bacterial community structure

**Fig. 2:**
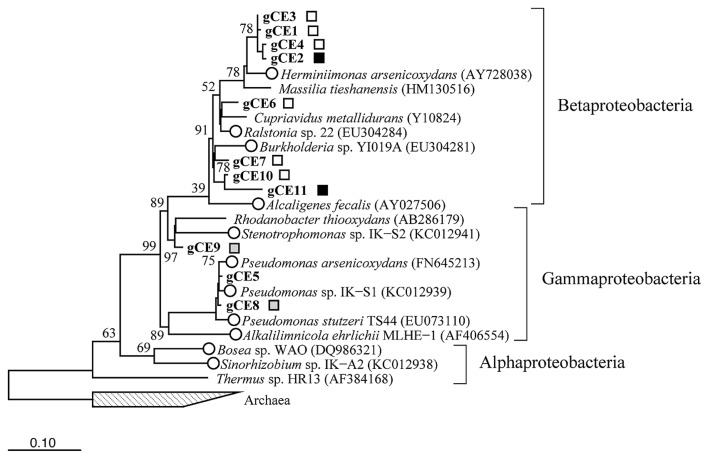
Neighbor-joining tree showing phylogenetic positions of 16S rRNA gene sequences (284 bp) from the DGGE analysis. The number in the sequence name following “gCE” corresponds to the DGGE band label in Fig. S3A. Bootstrap values (per 5,000 resamples) were shown on nodes. Open circle, As(III)-oxidizing bacteria; white, black, and gray squares indicate the DGGE band that commonly appeared among all treatments, only in the Sb(III)-tar treatment, or only in the As control treatment, respectively. Bar=0.1 substitutions per sequence position. Archaea were used as an outgroup.

In order to monitor the bacterial community structure established in soil columns during As(III) oxidation, a 16S rRNA gene-targeted DGGE analysis was conducted (Fig. S3A). DGGE banding patterns showed the emergence of some specific populations along with common populations among the different treatments (Fig. 2, Table 2). In particular, some specific bands only appeared in the As(III)-amended control treatment (*i.e.*, gCE 8 and 9 in Table 2) or Sb(III)-tar treatment (*i.e.*, gCE 2 and 11 in Table 2), but were absent from other treatments. The sequencing of prominent DGGE bands revealed that the 16S rRNA gene sequences of the commonly observed DGGE bands among all treatments (*i.e.*, gCE 1, 3, 4, 6, 7, and 10 in Fig. 2) belonged to *Betaproteobacteria*, closely related to the known As(III) oxidizer *Herminiimonas arsenicoxydans* (35) and heavy metal-resistant *Cupriavidus* sp. (34) (Table 2 and Fig. 2). The sequences detected in the Sb(III)-tar treatment only (*i.e.*, gCE 2 and 11 in Fig. 2) were related to the betaproteobacterial As(III) oxidizers, *Burkholderia* sp. (38) and *Herminiimonas* sp. (35). The sequences detected in the As(III)-amended control treatment only (*i.e.*, gCE 8 and 9 in Fig. 2) were closely related to the gammaproteobacterial As(III) oxidizers, *Pseudomonas* sp. IK-S1 and *Stenotrophomonas* sp. IK-S2, which were previously isolated from the same mine tailing soil used in the present study (18). Most of the 16S rRNA gene sequences identified in this study belonged to *Betaproteobacteria* and *Gammaproteobacteria*. Both of these phylogroups were also dominantly detected in soil-water environments under metal contamination (7, 10, 40, 42).

**Fig. 3:**
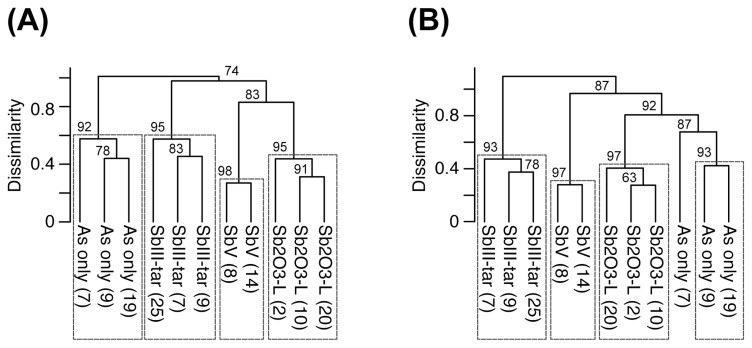
Multiple scaling bootstrap clustering analysis of DGGE banding patterns of 16S rRNA (A) and *aioA* (B) genes. DGGE bands representing >0.1% of the total intensity in each lane were used for this analysis. The number on the node indicates the *P*-value (%) calculated by 2,000 bootstrap resamplings. The numbers in parentheses indicate DGGE bands in Fig. S3. The rectangles indicate the supported cluster larger than 90% of the *P*-value.

The UPGMA clustering analysis of the 16S rDNA-based DGGE banding pattern (Fig. 3A) revealed significant similarities within each treatment (approximately unbiased *P*-value>90%). Furthermore, all three Sb co-amendment treatments showed more similar patterns than the As(III)-only treatment, indicating the potential establishment of populations tolerant to As and Sb. Consequently, co-amendments with various Sb chemical forms impacted the structure of bacterial populations.

### Arsenite oxidase gene (*aioA*) diversity

The arsenite oxidase gene, *aioA*, was successfully PCR amplified from all soil columns. The *aioA*-targeted DGGE analysis showed the establishment of a population with various *aioA* genotypes in each amendment (Fig. S3B). Similar to the 16S rDNA-based DGGE analysis, the *aioA*-banding pattern also indicated the emergence of some specific populations along with common populations among the treatments. Seven *aio*-DGGE bands were sequenced and all of these sequences were clustered with known *aioA* from *Alpha*- and *Gammaproteobacteria* (Table 2 and Fig. S4). Four of the *aioA* sequences obtained from Sb_2_O_3_-L (*i.e.*, gCX 2, 3, 4, 5, and 6 in Table 2) were closely related (96.2–99.6% nt identity) to the *aioA* sequence from *Pseudomonas* sp. CF161 (Fig. S4). Although only limited sequencing efforts were conducted, it still confirmed that *aio*-DGGE bands appeared to represent distinct *aio*-possessing populations in soil columns. More intensive sequencing efforts are needed in order to detect *aio* from other phylogroups identified in the 16S rRNA analysis (such as *Harminiimonas* sp.).

The cluster analysis conducted with *aio*-DGGE banding patterns also confirmed that variations in DGGE patterns among the different treatments were significantly larger than those observed within the same treatment (approximately unbiased *P*-value>90%; Fig. 3B). The DGGE profile of the Sb(III)-tar treatment was the most dissimilar from the other treatments, indicating the selection of distinct As(III)-oxidizing populations resistant to Sb(III) toxicity. The effects of Sb(III)-tar may include the influences of Sb(III) and tartrate, although tartrate did not exert any inhibitory effects on As(III)-oxidizing activity (Fig. S1). Previous studies showed that amendments with metal(loid)s shifted the bacterial community to exhibit higher resistance to metal(loid)s toxicity (21, 32, 49). Regarding As, a correlation was reported between the bioavailability of As and recovery of As(III)-resistant bacteria (49), and As(III)-resistant bacteria were selected due to the high toxicity of As(III) exerting a strong selective pressure (1, 20).

Collectively, our results demonstrated that the presence of different Sb chemical forms has a significant effect on the selection of distinct bacterial populations as well as As(III)-oxidizing bacterial populations in soil. Furthermore, additional contamination with Sb appeared to impose a stronger selective pressure on the soil bacterial community than a single As contamination, and, thus, also affected the redox transformation of contaminants in the natural environment.

### Abundance of 16S rRNA and *aioA* genes

**Fig. 4:**
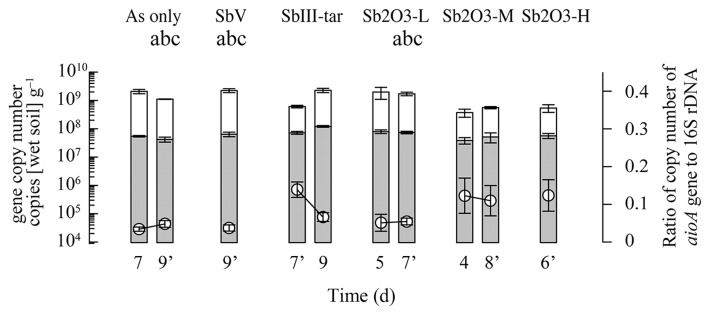
Abundance of 16S rRNA (white bars) and *aioA* (gray bars) genes during column experiments. The ratios of *aioA* to 16S rRNA gene copy numbers are shown in white circles. Error bars indicate the standard deviation of the mean values (*n*=3) of at least triplicate columns with triplicate qPCR technical replicates. The apostrophe shows samples applied to the Tukey’s post-hoc test, and results are expressed by the symbols. a: *P*<0.05 vs. Sb(III)-tar; b: *P*<0.05 vs. Sb2O3-H; c: *P*<0.05 vs. Sb2O3-M, respectively.

Bacterial abundance was examined by a qPCR analysis of 16S rRNA genes and ranged less than 10-fold (*i.e.*, between 2.9±0.8×10^8^ and 1.5±0.2×10^9^ copies g^−1^ soil) during a 4- to 9-d incubation among all treatments (Fig. 4). Bacterial abundance in the Sb_2_O_3_-M and Sb_2_O_3_-H treatments was significantly lower than that of other treatments, except Sb(III)-tar on day 7 (Tukey’s post-hoc test, *P*<0.05), which indicated that the potential toxicity of Sb(III) and antimony trioxide at higher concentrations inhibited bacterial growth. In the Sb(III)-tar treatment, bacterial abundance appeared to recover by day 9. Since the effects of Sb(III)-tar may be influenced by Sb(III) and tartrate, this recovery may be explained by the establishment of populations resistant to Sb(III) and/or capable of tartrate utilization as an additional carbon source (48).

The copy numbers of *aioA* genes ranged between 3.5±0.8×10^7^ and 1.0±0.07×10^8^ copies g^−1^ soil among the treatments (Fig. 4). This is similar to previously reported *aioA* copy numbers (8.1±1.3×10^6^ to 1.7±0.1×10^7^ copies g^−1^ soil) in As(III)-amended soil columns showing similar As(III)-oxidizing activities (128~158 μmol L^−1^ d^−1^) (23). The Sb(III)-tar treatment contained significantly more abundant *aioA* genes than the other treatments (Tukey’s post-hoc test, *P*<0.05), except for Sb_2_O_3_-L (*P*>0.2). The relative abundance of *aioA* genes, calculated as ratios of *aioA* gene copies to 16S rRNA gene copies, were compared among the treatments at the time point when the maximum As(III) oxidation rate was reached (Fig. 4). These ratios ranged between 0.03 and 0.14, which were consistent with the previously reported ratios of 0.01 to 0.14 in As-polluted waters (39) and 0.01 to 0.04 in As(III)-amended soil columns (23). Among the treatments, a significantly higher (Tukey’s post-hoc test, *P*<0.05) relative abundance of *aioA* was detected in Sb(III)-tar, Sb_2_O_3_-M, and Sb_2_O_3_-H co-amendments, consistent with potential responses to high Sb(III) toxicity in these treatments. This result indicates that the *aio*-possessing population established under high Sb toxicity tolerates Sb. Antimony toxicity is known to induce oxidative stress via a similar mechanism to that of As (2, 16). Since As(III)-oxidizing bacteria are capable of tolerating As toxicity (14), they may also tolerate Sb toxicity using similar mechanisms. Indeed, the *ars* genes, coding for arsenic resistance, are also considered to be involved in antimony resistance (13).

Under Sb co-amendment conditions, there did not appear to be any correlation between *aioA* gene abundance and the rates of As(III) oxidation (Fig. 1 and 4). In the Sb(III)-tar co-amendment, the As(III) oxidation rate decreased to 30% by day 7, while it contained more abundant *aioA* genes than other treatments with higher As(III) oxidation activities. Furthermore, in all three Sb_2_O_3_ co-amendments with different Sb_2_O_3_ concentrations, similar As(III) oxidation rates were observed (Fig. 1D–F) despite differences in *aioA* gene densities and bacterial community structures. In previous studies, *aio* gene densities were found to correlate with As pollution levels in surface waters (39) and with rapid As(III) oxidation in a high-As hot spring water (33), however, the effects of As contamination may be significantly complicated by the presence of antimony (4). Consequently, the co-contamination with As and Sb may influence the microbial community structure and As(III)-oxidizing activity by multiple mechanisms, which may involve the specific inhibition of As(III) oxidation by various Sb forms, the regulation of *aio* gene expression (19), and the selection of distinct populations with different specific As(III) oxidation rates (5).

## Conclusions

In the present study, the effects of co-contamination with As and Sb on soil microbial As(III) oxidation was shown to be influenced by the chemical form of Sb. Microbial As(III) oxidation was markedly inhibited by co-contamination with Sb(III)-tartrate, but not with the mineral oxide form of Sb(III), Sb_2_O_3_, or Sb(V). The presence of Sb species shifted the microbial community structure, resulting in the selection and enrichment of adapted As(III) oxidizers with potentially higher tolerance to As and Sb toxicity. The abundance of *aio* genes may not correlate with the soil microbial As(III) oxidation rate under co-contamination conditions, possibly due to the complex synergistic and antagonistic inhibitory effects of As and Sb on the microbial community. Collectively, the present results demonstrated that co-contamination may affect microbial community diversity and composition, as well as the redox transformation of contaminants *in situ*.

## Supplementary Material



## Figures and Tables

**Table 1:** Summary of soil column treatments.

Amendment	Soil column treatments^a^					

	As only	Sb(V)	Sb(III)-tar	Sb2O3-L	Sb2O3-M	Sb203-H
Arsenic	As(III)	As(III)	As(III)	As(III)	As(III)	As(III)
(mM)	0.2	0.2	0.2	0.2	0.2	0.2
Antimony	NA^b^	Sb(V)	Sb(III)-tar	Sb_2_O_3_	Sb_2_O_3_	Sb_2_O_3_
(mM)	0	0.4	0.15	4.1^c^	9.6^c^	46^c^

a Concentrations of As and Sb added to the influent are shown. As(III), Sb(V), Sb(III)-tar, and Sb2O3 were added as forms of NaAsO_2_, K(Sb[OH]_6_), C_8_H_4_K_2_O_12_Sb_2_, and Sb_2_O_3_, respectively.

b NA: not applied.

c Calculated as a concentration of Sb(III) in the soil solution added as Sb_2_O_3_ to the column.

**Table 2:** Closest relatives of 16S rDNA and *aioA* gene fragments detected in column experiments.

DGGE band^a^	Closest relatives in GenBank^b^				Band appearance in each treatment^c^			
	
	Phylogenetic group^d^	Strain and Species	Accession no.	% Similarity	As only	Sb(III)-tar	Sb2O3-L	SbV
16S rDNA								
gCE1	*Betaproteobacteria*	*Massilia lutea* strain 101	NR_043310	100	+	+	+	+
gCE2	*Betaproteobacteria*	*Janthinobacterium agaricidamnosum* NBRC 102515	HG322949	99		+		
gCE3	*Betaproteobacteria*	*Massilia albidiflava* strain 45	NR_043308	100	+	+	+	+
gCE4	*Betaproteobacteria*	*Janthinobacterium agaricidamnosum* NBRC 102515	HG322949	99	+	+	+	+
gCE5	*Gammaproteobacteria*	*Pseudomonas* sp. NL6	KJ819580	100	+	+	+	
gCE6	*Betaproteobacteria*	*Cupriavidus* sp. strain SaCRH15	JX233515	99	+	+	+	+
gCE7	*Betaproteobacteria*	*Burkholderia metallica* strain g33	KM019869	100	+	+	+	+
gCE8	*Gammaproteobacteria*	*Pseudomonas* sp. strain CY63	JF933911	99	+			
gCE9	*Gammaproteobacteria*	*Rhodanobacter* sp. strain 2D4	KC987462	100	+			
gCE10	*Betaproteobacteria*	*Burkholderia* sp. strain ES3-67	KJ878640	100	+	+	+	+
gCE11	*Betaproteobacteria*	*Burkholderia* sp. strain ES3-67	KJ878640	99			+	
*aioA*								
gCX1	*Gammaproteobacteria*	*Achromobacter* sp. strain 38AGIII	HQ449654	93	+	+	+	+
gCX2	*Gammaproteobacteria*	*Pseudomonas* sp. strain 3AAV	HQ449658	91			+	
gCX3	*Gammaproteobacteria*	*Pseudomonas* sp. strain 3AAV	HQ449658	91	+	+	+	
gCX4	*Gammaproteobacteria*	*Pseudomonas* sp. strain 3AAV	HQ449658	91	+	+	+	
gCX5	*Gammaproteobacteria*	*Pseudomonas* sp. strain 3AAV	HQ449658	91	+	+	+	
gCX6	*Gammaproteobacteria*	*Bacillus flexus* strain ADP-25	JX188049	90		+	+	
gCX7	*Alphaproteobacteria*	*Bosea* sp. strain L7506	EF637043	94	+	+	+	

a DGGE band names correspond to those in Fig. 2 and S4.

b The closest cultivated organism is listed.

c DGGE bands present in each treatment were shown as “+”.

d See Fig. 2 and S4 for more details.
