# Supplementary material for: Influence of the Chemical Form of Antimony on Soil Microbial Community Structure and Arsenite Oxidation Activity
Source: Microbes Environ. 2018 Jun 9;33(2):214–21. doi: 10.1264/jsme2.ME17182 (PMC6031390; doi:10.1264/jsme2.ME17182)

Supplemental Online Material:

Title: The influence of chemical form of antimony on soil microbial community structure and arsenite oxidation activity

Authors: Kataoka, T., Mitsunobu, S., and N. Hamamura

Supplemental figure S1.
The production of As(V) resulted from As(III) oxidation and concentration of total As in effluent from the column amended with As(III) and potassium-L-tartrate monobasic. Closed circles represent the percentage of As(V) produced from oxidation of As(III) to total As concentration, and open circles represent total As concentrations (mM) in effluent. Each point represents the mean of at least triplicate soil columns; the error bars represent the standard error, and where absent, error bars are smaller than symbol size.

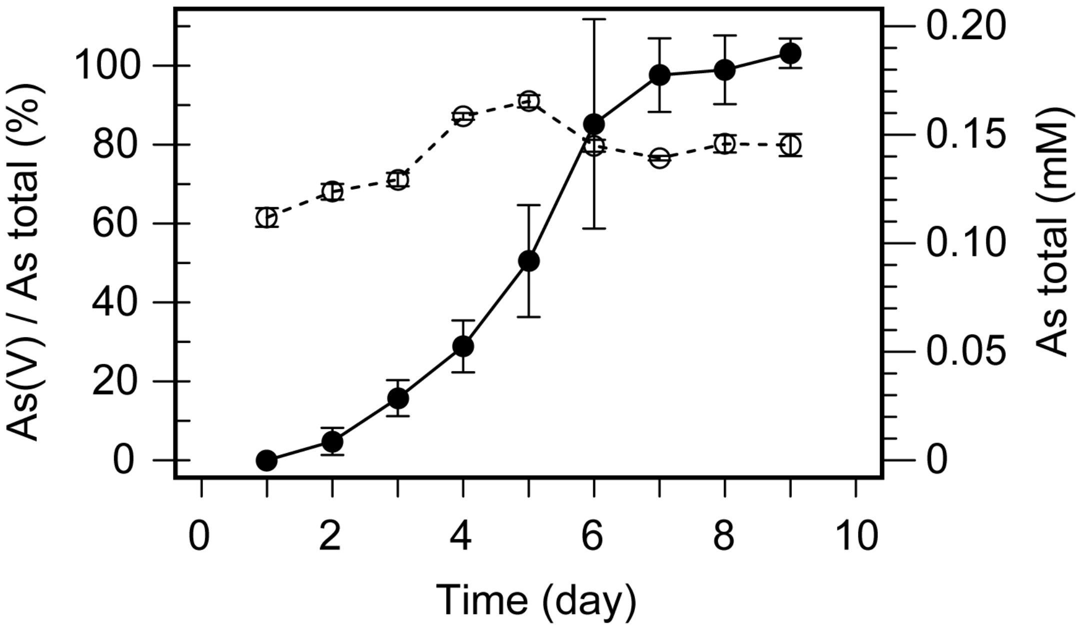

Supplemental figure S2.

Concentrations of total soluble Sb (open triangles) and Sb(V) (closed triangles) in effluent from column experiments. The experimental conditions in each plot (A) – (F) correspond to Fig.1 and Table 1. Each point represents the mean of at least triplicate soil columns; the error bars represent the standard error, and where absent, error bars are smaller than symbol size.

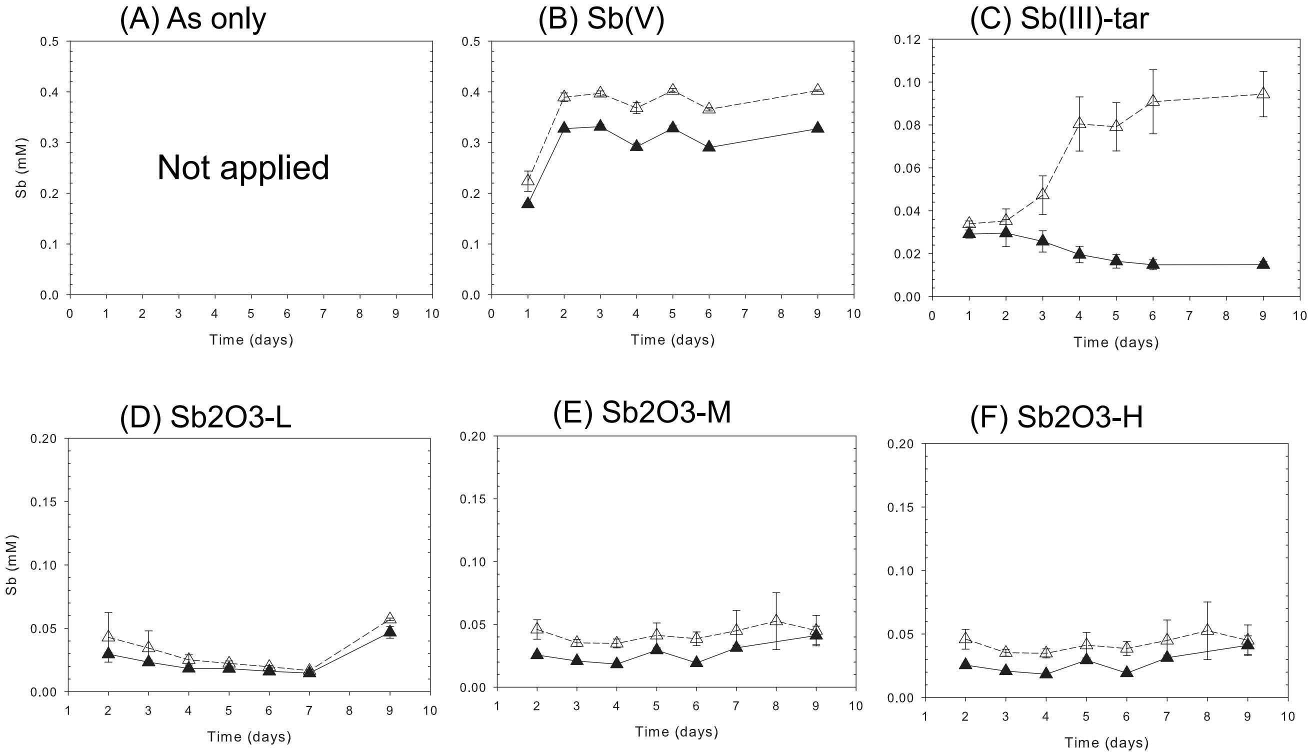

Supplemental figure S3.
DGGE profiles of 16S rRNA (A) and *aioA* (B) gene fragments from column experiments. The nucleotide sequences of the labeled bands were determined and described further (Table 2).

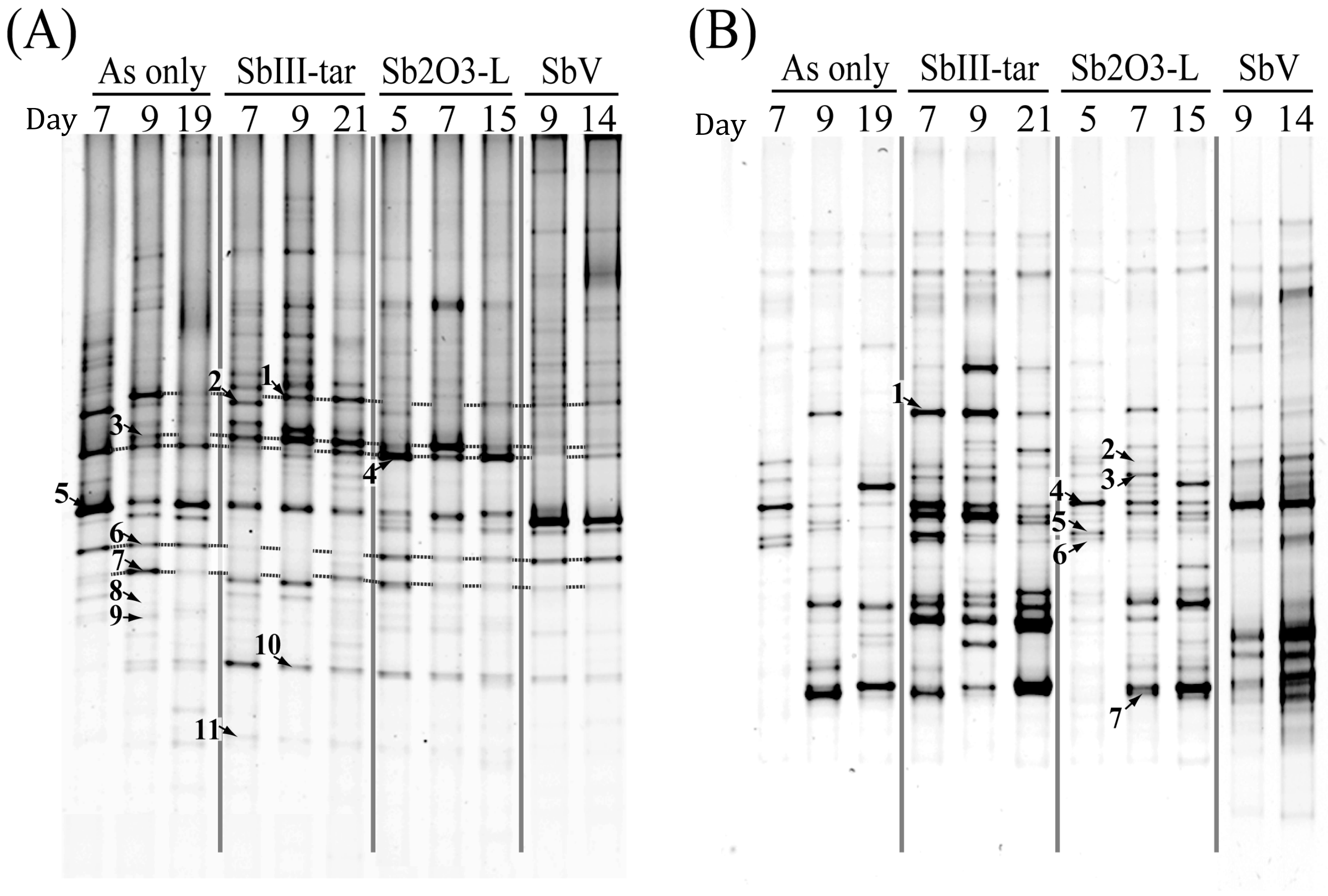

Supplemental figure S4.

Neighbor-joining tree of the deduced amino acid sequences encoded by the putative *aioA* genes (161 aa) obtained from DGGE analysis. Number in sequence name, following “gCX”, corresponds to the DGGE band label in Fig. S3B. Bootstrap values (per 2000 trials) more than 50% are shown. Bar indicates substitutions per sequence position. Two sequences of *aioA* gene from *Chloroflexus* spp. were used as out group.

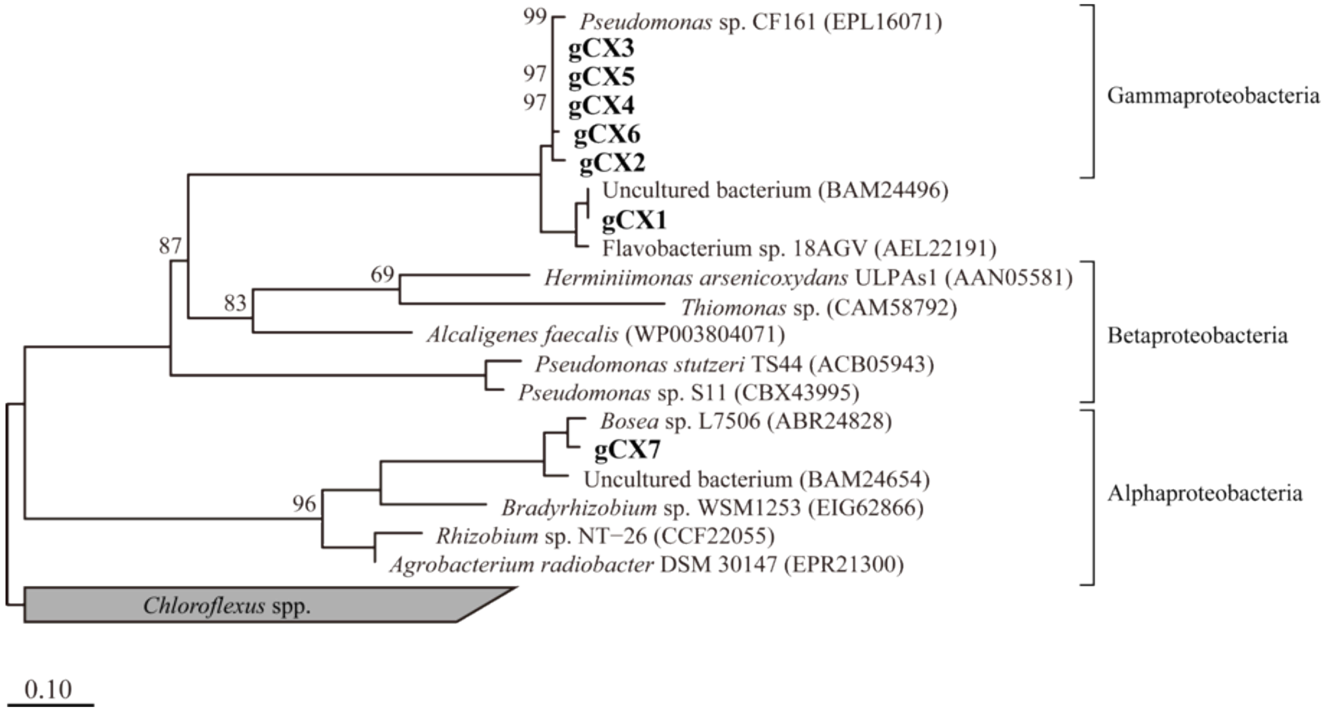

Supplement: Supplementary file 1 [file 33_214_s1.pdf]
